# The molecular engineering, synthesis and photovoltaic studies of a novel highly efficient Ru(ii) complex incorporating a bulky TPA ancillary ligand for DSSCs: donor *versus* π-spacer effects†

**DOI:** 10.1039/c9ra06150a

**Published:** 2020-01-02

**Authors:** Islam M. Abdellah, Ahmed El-Shafei

**Affiliations:** Department of Chemistry, Faculty of Science, Aswan University Aswan 81528 Egypt; Polymer and Color Chemistry Program, Department of TECS, North Carolina State University Raleigh 27606 USA Ahmed_El-Shafei@ncsu.edu

## Abstract

A novel Ru(ii) complex, denoted as IA-7, incorporating a bulky donor antenna, was synthesized and characterized as a promising inexpensive alternative to conventional p–n junction solar cells to study the influence of a bulky donor antenna on the light harvesting efficiency (LHE), ground and excited state oxidation potentials and total conversion efficiency of sunlight to electricity (% *η*) for dye-sensitized solar cells (DSSCs), and the device performance was compared to devices with MH-12 and MH-13 dyes. The incorporation of the bulky donor enriched triphenylamine (TPA) antenna resulted in a considerable increase in *J*_SC_ and *η* values for DSSCs, where IA-7 outperformed MH-12 and MH-13 in terms of the total conversion efficiency and achieved a power conversion efficiency (*η*) of 8.86% under full sunlight irradiation (100 mW cm^−2^), compared to 8.09% for MH-12 and 8.53% for MH-13, which can be ascribed to the high open circuit voltage (*V*_OC_) of IA-7. Molecular engineering utilizing DFT/TD-DFT was employed to calculate the electronic properties of IA-7, including the HOMO/LUMO isosurfaces, the lowest singlet–singlet electronic transitions (*E*_0–0_), and the ground and excited state oxidation potentials, which were in ideal agreement with the empirical results. The electronic distribution of IA-7 indicated that the HOMO is delocalized not only on Ru and NCS, but also on the substituted TPA, and the LUMO is delocalized over 4,4′-bipyridyl dicarboxylic acid.

## Abstract 2

The IA-7 complex was applied in DSSCs.

## Graphical abstract

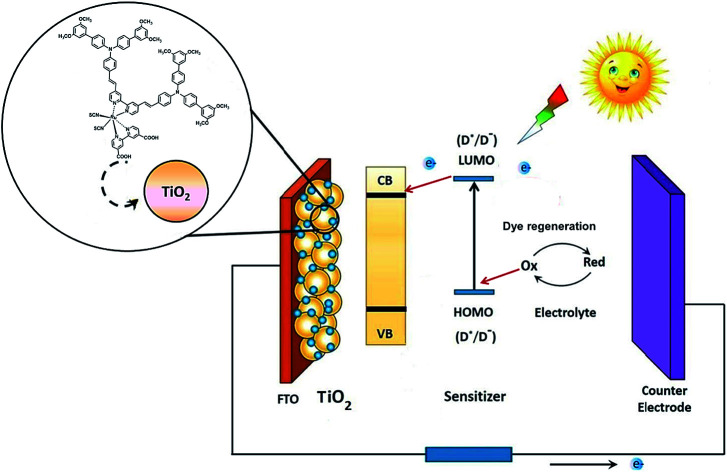

## 1. Introduction

One of the huge difficulties that mankind faces is exchanging petroleum derivatives with sustainable power sources, while combating the growing energy requirements and exhaustion of energy resources. This challenge must be answered with low-cost, clean and generously available crude materials. The sun is a clean and cheap energy source, and supports life on earth. Accordingly, harvesting solar energy with photovoltaic innovations gives the impression of being the main logical response to the energy challenge. One of the fascinating advancements is DSSCs, which were investigated by O'Regan and Grätzel.^1^ DSSCs have attracted all-inclusive academic and business interest because of their extraordinary performance under diffuse light conditions and low illumination levels, and their independence of the incident light angle.^2–10^ DSSCs have numerous advantages compared to silicon-based solar cells, including transparency, low cost, and high-power conversion efficiencies under low light levels and artificial light sources.^11^ Moreover, the working standards of DSSCs include the ingestion of a photon by the sensitizer, and the change to an energized state, which injects an electron into the semiconductor conduction band. The injected electron travels through the mesoporous semiconductor arrangement of particles to land at the back-collector anode and then through the external circuit to the cathode to diminish the oxidized iodide and recover the sensitizer. Some negative responses prompt a decline in the cell efficiency; for example, recombination of the injected electrons either with the oxidized sensitizer or with the redox electrolyte.^12^ Subsequently, accomplishing higher efficiency devices is reliant on the optimization and compatibility of all of the components in the device at the same time, specifically the semiconductor film surface area onto which the dye can be adequately absorbed.^13–15^ Ru(ii)-based complexes have been shown to be viable sensitizers for DSSCs in light of their uncommon metal to ligand charge transfer (MLCT) transitions, unique excited state photostability and photophysics. The most efficient ruthenium constructs for DSSCs were made by the Grätzel group; for example, N719, N3 and black dyes.^16^ The exceptional light harvesting and durability features of these photosensitizers are based on the MLCT transition, through which the photoelectric charge is moved to the TiO_2_ at a quicker rate than electron recombination with the oxidized dye molecules, instead of moving through the circuit.^17–20^ Clearly, research on dye preparation for DSSC applications is becoming increasingly sophisticated and many ruthenium-based structures have been reported.^21,22^ The most favorable strategy for constructing effective sensitizers for DSSCs is to molecularly build the sensitizer to exhibit predominant light harvesting, while maintaining all of the thermodynamic and kinetic requirements for electron injection, hole replenishment and charge recombination suppression.

**Scheme 1:**
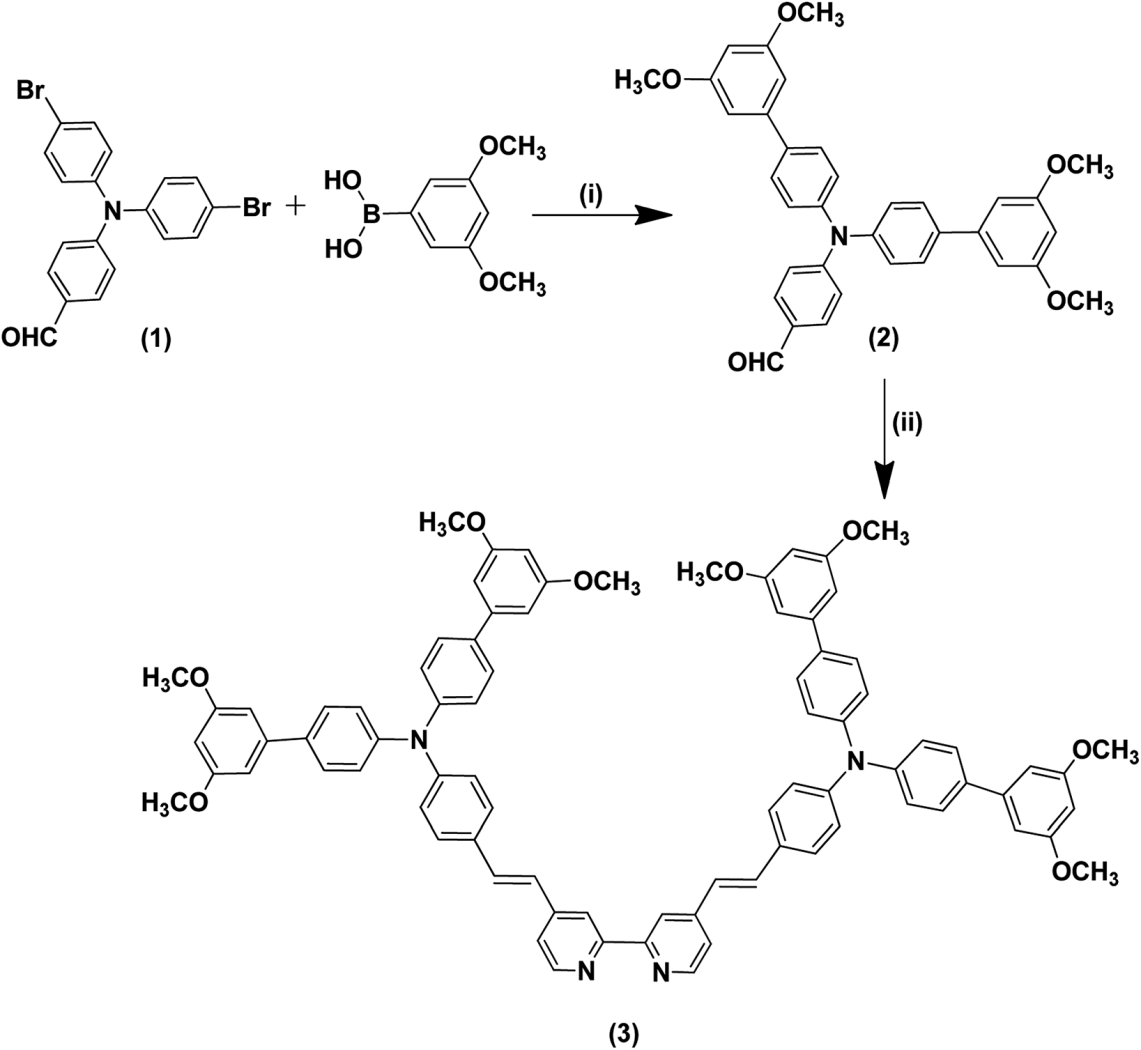
Synthetic routes for the bulky antenna ligand (3). (i) Aliquat-336, K_2_CO_3_, tetrakis(triphenylphosphine)palladium(0), H_2_O, THF, 90 °C, 4-boronobenzoic acid, and 5-formyl-2-thiopheneboronic acid; (ii) 4,4′-dimethyl-2,2′-dipyridyl, chlorotrimethylsilane, pressure tube, 115 °C.

**Scheme 2:**
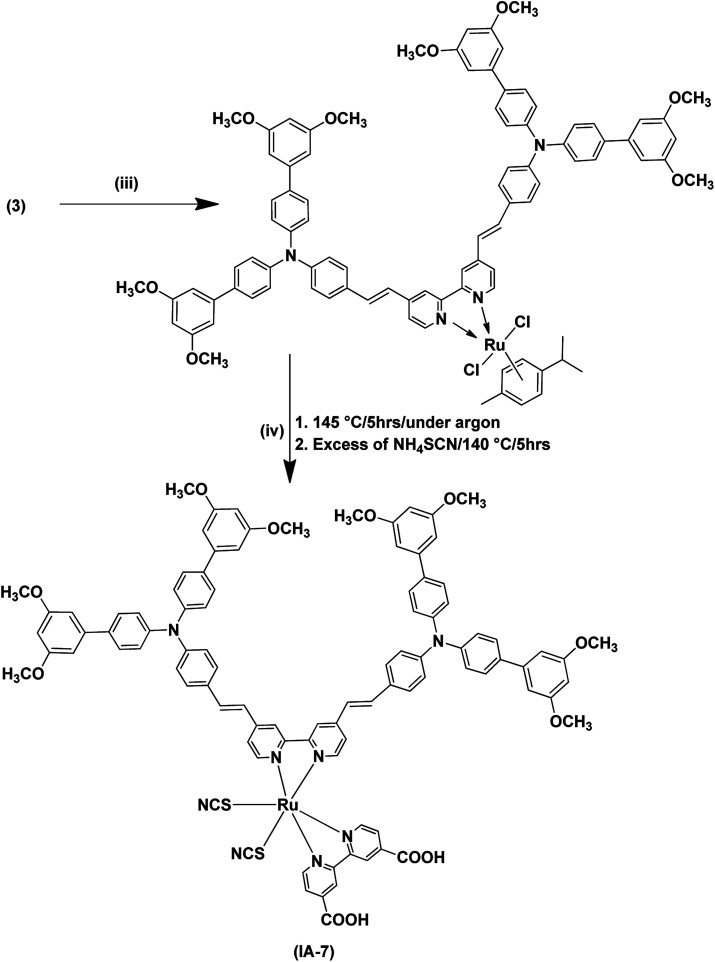
Synthetic routes for the dye IA-7. (iii) Dichloro-(*p*-cymene)-ruthenium(ii) dimer, anhydrous DMF, 95 °C, 5 h, under argon gas; (iv) 2,2′-bipyridinyl-4,4′-dicarboxylic acid.

Herein we report the preparation of a new complex (IA-7) based on a bulky triphenylamine ancillary ligand to form a highly light harvesting and effective donor, which is directly linked to Ru metal. The newly designed IA-7 has been synthesized in three steps, starting with the preparation of 4-(bis(3′,5′-dimethoxy-[1,1′-biphenyl]-4-yl)amino) benzaldehyde through Suzuki–Miyaura coupling, followed by Knoevenagel condensation reaction with 4,4′-dimethyl-2,2′-bipyridine to form the required ancillary ligand and finally target dye formation in a one-pot three-step reaction as shown in Scheme 2. The synthetic routes and structure of IA-7 are depicted in Schemes 1 and 2. Moreover, the photovoltaic and photophysical properties of IA-7 incorporating a bulky donor ancillary ligand were compared to the well-known photosensitizers MH-12 and MH-13 (ref. 23) incorporating π-spacer ancillary ligands to illustrate the effect of donor and π-spacer ancillary ligands on the PCE.

**Fig. 1:**
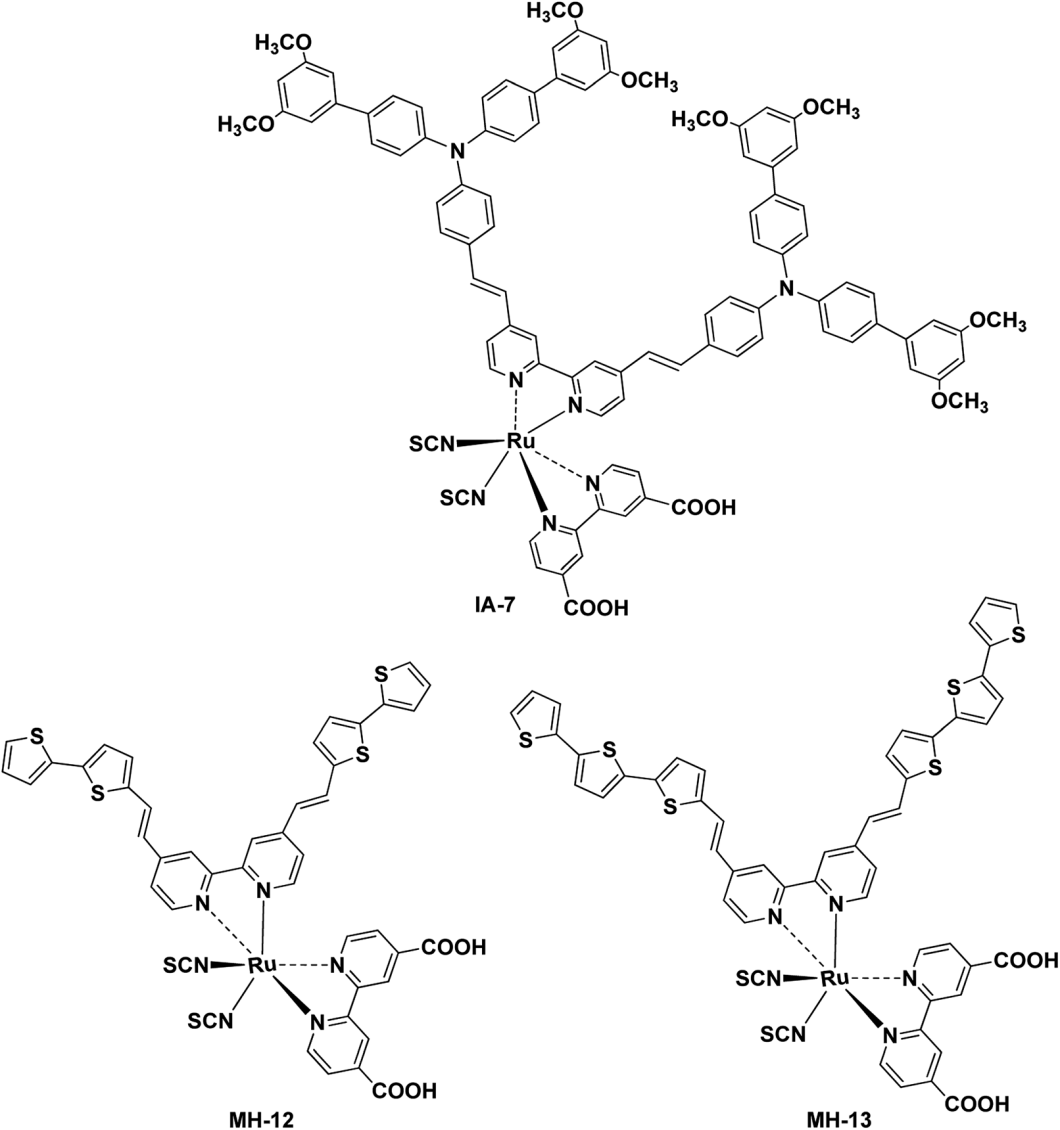
Chemical structures of the IA-7, MH-12, and MH-13 complexes for DSSCs.

Fig. 1 depicts the chemical structure of IA-7 carrying a bulky donor ancillary ligand, along with the MH-12 and MH-13 sensitizers carrying π-spacer ancillary ligands. The newly synthesized molecules were characterized using different analytical methods; for example, ^1^H-NMR, FT-IR and high-resolution mass spectroscopy analysis. Their electronic properties and band gap were resolved from UV-Vis absorption and fluorescence emission studies. Furthermore, the energetics of the GSOP-ESOP thermodynamic parameters was determined from the cyclic voltammetry technique. Finally, the dyes were used as photosensitizers towards the fabrication of DSSCs to evaluate their photovoltaic parameters, such as IPCE, PCE, *J*_SC_, *V*_OC_, and FF. Also, EIS studies were performed to evaluate the recombination resistance, the transport resistance, the capacitance of electron accumulation and measurements of electron lifetimes in the fabricated DSSCs.

## 2. Experimental

### 2.1. Materials and methods

The starting materials, including *N*-bromosuccinimide, 4-(*N*,*N*-diphenylamino)benzaldehyde, (3,5-dimethoxyphenyl)boronic acid, aliquat-336, K_2_CO_3_, Pd(PPh_3_)_4_, 4,4′-dimethyl-2,2′-dipyridyl and trimethylsilane, were purchased from Sigma-Aldrich, Alfa Aesar and Ark Pharm companies. In addition, 2,2′-bipyridinyl-4,4′-dicarboxylic acid and the MH-12 and MH-13 photosensitizers were synthesized according to the reported procedure^23,24^ (see ESI†). All required solvents were ordered from Fischer Scientific. A Bruker advance 400 MHZ was used to obtain the ^1^H-NMR spectra, applying DMSO-*d*_6_ and TMS as an internal standard for chemical shift calibration. A Bruker alpha spectrophotometer was used for FTIR spectroscopy analysis. The mass spectra were acquired on a Thermo Scientific EXACTIVE (ESI-MS) spectrometer. The UV-visible and fluorescence spectra were obtained utilizing SPECORD S600 and Horiba Fluromax-4 spectrophotometers.

### 2.2. Synthesis and structural characterization

#### 2.2.1. 4-(Bis(4-bromophenyl)amino)benzaldehyde (1)

In a 100 ml three necked flask, 4-(*N*,*N*-diphenylamino) benzaldehyde (1 mmol, 1.366 g) was added to DMF (50 ml), under a nitrogen atmosphere, and the reaction mixture was cooled to −5 °C with an ice bath. While the solution was stirred in the dark, a dropping funnel was used for the drop-wise addition of *N*-bromo succinimide (2 mmol, 1.77 g) dissolved in DMF (15 ml), and the mixture was stirred for 2 hours at 0 °C. After 2 hours, the solution was stirred overnight at 25 °C. The reaction mixture was extracted by equal amounts of water and chloroform. The organic layer was separated using a funnel, dried over MgSO_4_ and evaporated under pressure to form a yellow solid, which was purified *via* silica gel chromatography (CHCl_3_ : Hex.) (3 : 1) and crystallized in methanol. Yield (97.41%), melting point (mp) 161 °C. FT-IR (cm^−1^): 2832 (C–H aldehydic), 1682 (C=O), 1573 (C=C aromatic), 1270 (C–N aromatic amine), 724 (C–Br). ^1^H NMR (600 MHz, chloroform-*d*, *δ*): 9.75 (s, 1H), 7.73–7.67 (m, 2H), 7.33 (m, 6H), 7.05–6.99 (m, 4H). MS: *m*/*z* (C_19_H_13_Br_2_NO) found = 431.942 (calcd 431.941 For [M + H]^+^) with an error of Δ*M* = 2.23 ppm.

#### 2.2.2. 4-(Bis(3′,5′-dimethoxy-[1,1′-biphenyl]-4-yl)amino)benzaldehyde (2)

4-(Bis(4-bromophenyl)amino)benzaldehyde (0.431 g, 1 mmol) and (3,5-dimethoxyphenyl)boronic acid (0.4 g, 2.2 mmol) were dissolved in 40 ml of THF and two drops of aliquat-336 solution were added. The reaction mixture was then degassed under argon for 30 minutes and tetrakis(triphenyl-phosphine)palladium(0) (0.068 g, 0.06 mmol) was added, followed by the addition of K_2_CO_3_ solution (2 M, 5 ml), and the mixture was stirred for 10 hours at 85 °C. The reaction was tracked by TLC until completion. The resulting mixture was cooled and extracted with methylene chloride, and the organic layer was dried over Na_2_SO_4_, and filtered. After removing the solvent, the compound was purified by silica gel chromatography by using chloroform as an eluant to give a yellow solid. Yield (61%), melting point (mp) 174 °C. FT-IR (cm^−1^): 2954 & 2834 (C–H, alkane), 1687 (CO),1306 (C–N, aryl). ^1^H NMR (600 MHz, chloroform-*d*, *δ*): 9.74 (s, 1H), 7.66–7.60 (m, 2H), 7.45–7.40 (m, 4H), 7.19–7.09 (m, 6H), 7.09–7.04 (m, 2H), 6.52–6.50 (m, *J* = 7.9 Hz, 4H), 3.42 (s, 12H). ESI-FTMS: *m*/*z* (C_35_H_31_NO_5_): found = 546.22794 (calcd 546.2275 for [M + H] ^+^).

#### 2.2.3. *N*,*N*′-(((1*Z*,1′*E*)-[2,2′-Bipyridine]-4,4′-diylbis(ethene-2,1-diyl))bis(4,1-phenylene))bis(*N*-(3′,5′-dimethoxy-[1,1′-biphenyl]-4 yl)-3′,5′-dimethoxy-[1,1′-biphenyl]-4-amine) (3)

Ligand 3 was synthesized in a pressurized glass tube containing 4,4′-dimethyl-2,2′-bipyridine (0.184 g, 1 mmol), 4-(bis(3′,5′-dimethoxy-[1,1′-biphenyl]-4 yl)amino) benzaldehyde (2) (0.54 g, 1 mmol), and 0.76 ml of chlorotrimethylsilane (6 mmol) in 60 ml of anhydrous DMF. The tube was sealed well with the cap, the reaction temperature was raised to 110 °C and the reaction was allowed to run for 48 hours with continuous stirring, during which time the color of the reaction mixture changed to dark orange. After 48 hours, the tube was cooled, the pressure of the tube was released, and the solvent was evaporated using a rotary evaporator leaving a dark orange crude product, which precipitated by adding 150 ml of icy water. Finally, vacuum filtration of the antenna ligand was performed, followed by washing with plenty of water and toluene. The crystals were collected and dried overnight at 50 °C. The antenna ligand was recrystallized from methanol to form pure dark red crystals. Yield (79%), melting point (mp) 190 °C. FT-IR (cm^−1^): 2936&2833 (C–H, alkane), 1608 (C=N), 1293 (C–N, aryl). ^1^H NMR (600 MHz, chloroform-*d*, *δ*): 9.00–8.94 (m, 2H), 8.12 (d, *J* = 5.0 Hz, 1H), 8.09–8.02 (m, 3H), 7.68–7.62 (m, 2H), 7.58–7.51 (m, 3H), 7.49–7.29 (m, 15H), 7.27–7.18 (m, 6H), 7.12 (t, *J* = 2.0 Hz, 1H), 7.00–6.91 (m, 2H), 6.74–6.55 (m, 6H), 6.52 (t, *J* = 2.0 Hz, 1H), 6.38–6.36 (m, 2H), 6.27 (t, *J* = 2.0 Hz, 1H), 5.88 (t, *J* = 2.0 Hz, 1H), 3.85 (s, 24H). ESI-FTMS: *m*/*z* (C_82_H_70_N_4_O_8_) found = 1239.522 (calcd 1239.526 for [M + H] ^+^) with an error of Δ*M* = 0.812 ppm.

#### 2.2.4. Synthesis of photosensitizer IA-7

Complex IA-7 was synthesized in one pot using a three-step reaction. The reactions were carried out in a 150 ml flask. The flask was charged with 40 ml anhydrous DMF, dichloro-(*p*-cymene)-ruthenium(ii) dimer (0.3 g, 0.5 mmol) and antenna ligand 3 (1.23 g, 1 mmol). The mixture was stirred using a magnetic stirrer bar under argon at 90 °C for 6 h. Then, 2,2′-bipyridyl-4,4′-dicarboxalic acid was added (0.244 g, 1 mmol), the temperature was raised to 150 °C, and the reaction was allowed to keep running for 5 hours. After 5 hours, an excess of NH_4_NCS (0.5 g) was added, and the reaction mixture was permitted to keep running for a further 4 h at 140 °C. The reaction mixture was chilled off to room temperature and DMF was removed utilizing a rotary evaporator. Ice was added to the flask and a black solid was precipitated. The solid was filtered, washed with diethyl ether, and dried. After drying, the dye was dissolved in methanol with the addition of a few drops of tetrabutylammonium hydroxide (TBAOH) until the pH of the solution became slightly basic, and then it was run through a column containing silica gel. The violet main band was collected and acidified using 0.1 M HCl to reduce the pH to 2.0 and allowed to precipitate for 48 hours in a refrigerator. The precipitate was then filtered, and washed well with water to bring the pH to neutral. The pure dye was then dried overnight and collected as dark color crystals. Yield (69%), melting point (mp) 267 °C. FT-IR (cm^−1^): 3409 (OH), 2935 & 2834 (C–H, alkane), 2101 (NCS), 1724 (C=O), 1586 (C=N), 1278 (C–N, aryl).^1^H-NMR (600 MHz, DMSO-d_6_, *δ*): 9.10–9.03 (m, 2H), 8.74 (d, *J* = 7.5 Hz, 2H), 8.65 (d, *J* = 1.5 Hz, 2H), 7.38–7.32 (m, 4H), 7.36–7.15 (m, 7H), 7.08–7.00 (m, 2H), 6.96–6.87 (m, 5H), 6.74 (dd, *J* = 7.4, 1.5 Hz, 1H), 6.66 (t, *J* = 1.9 Hz, 2H), 6.29 (dd, *J* = 7.5, 1.4 Hz, 1H), 3.85 (s, 12H). ESI-FTMS: *m*/*z* (C_96_H_78_N_8_O_12_S_2_Ru) found = 1701.432 (calcd 1702.433 for [M + H]^+^) with an error of Δ*M* = 1.40 ppm.

## 3. Results and discussion

### 3.1. Synthesis and characterization

The synthetic pathways of the Ru(ii) complex (IA-7) are portrayed in Schemes 1 and 2. The synthesis was started by the preparation of aldehyde 2 utilizing C–C Suzuki coupling interaction, followed by the preparation of bulky antenna ligand 3 with the help of a Knoevenagel condensation reaction (Scheme 1). The synthesized aldehyde and the antenna ligand were purified and recrystallized.

In the final step, the target Ru(ii) complex (IA-7) was obtained by following a one-pot three-step reaction protocol, wherein the precursor antenna (3) was reacted with dichloro-(*p*-cymene)-ruthenium(ii) dimer, and then 2,2′-bipyridyl-4,4′-dicarboxalic and ammonium thiocyanate to form the target complex (IA-7) (Scheme 2). All of the new compounds and the target sensitizer were purified and recrystallized utilizing column chromatography techniques. The structures of the newly synthesized dyes and their intermediates were confirmed by various spectral techniques as shown in Fig. S1–S14 (see ESI†).

### 3.2. Photophysical and electrochemical studies

**Fig. 2:**
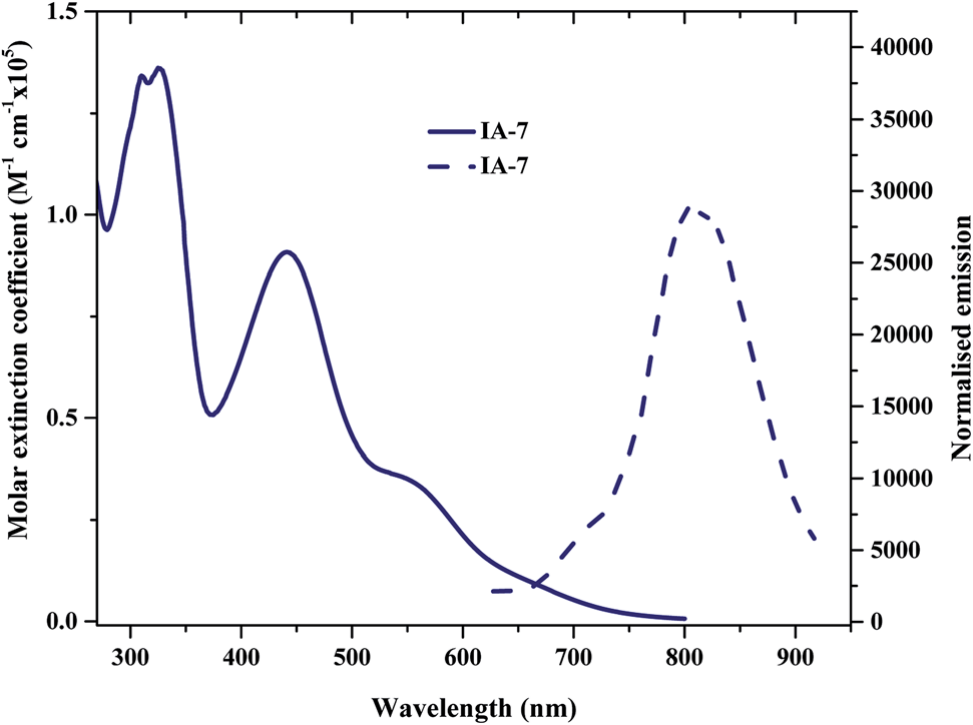
The normalized absorption and emission spectra of IA-7 recorded in CH_2_Cl_2_ (2 × 10^−5^ M).

The UV-Vis absorption (emission) spectra of IA-7 were measured in methylene chloride (2 × 10^−5^ M) solution. The spectra are depicted in Fig. 2 and the corresponding data are outlined in Table 1. The obtained optical properties for the photosensitizers MH-12 and MH-13 were compatible with the reported data.^23^ From the UV-Vis absorption spectra, all of the dyes possess three distinctive absorption bands. The band in the region of 310–320 nm is attributed to π–π* electronic transition of the bipyridine ligand, while the peak corresponding to wavelengths in the region of 400–445 nm can be ascribed to ligand-to-ligand charge transfer (LLCT) mixed with metal-to-ligand charge transfer (MLCT) (πd–π*), and the broad peak at 545–565 nm corresponding to the longest wavelength can be credited to metal-to-ligand charge transfer (MLCT) (πd–π*), which corresponds to electron transition from the HOMO energy level to the LUMO energy level.

**Table 1:** Comparison of the optical and electrochemical properties of the IA-7 complex in (2 × 10^−5^ M) methylene chloride solution^a^

Dye	*λ*_max_ (nm)	*ε*_max_ (M^−1^ cm^−1^)	Stokes shift (nm)	*E*_max_ (nm)	*I* (nm)	*E*_0–0_^*b*^ (eV)	GSOP (eV)	ESOP (eV)
IA-7	561 (d–π*)	35 841	245	806	659	1.88	−5.46	−3.58

a *λ*_max_: maximum absorption wavelength, *ε*_max_: molar extinction coefficient maxima, *E*_max_: wavelength of maximum emission, *I*: intersection of the absorption and emission spectra, and *E*_0–0_^*b*^: experimental optical band gap.

The UV-Vis spectra of all of the complexes are characterized by metal–ligand internal charge transfer transitions in the visible region from 400–700 nm. The molar extinction coefficients of these bands are in the following order: IA-7 > MH-13 > MH-12. It is clear from the UV-Vis absorption spectra that the peak positions of the lowest energy MLCT bands of the MH-12 and MH-13 sensitizers are slightly blue shifted by 8 nm and 7 nm, respectively, when compared with complex IA-7, and the molar extinction coefficient of the new complex is higher. The higher extinction coefficient of the IA-7 complex is attributed to presence of the 4,4′-triphenyl amine-2,2′-bipyridine ligand, which contains extended π-conjugation of the phenyl groups with substituted methoxy groups and the directionality of the excited state by perfect tuning of the ligand LUMO energy level with the donating groups. As a result, the strong electron donor ancillary ligand nature in IA-7 is responsible for the increase in molar extinction coefficient in the visible region as compared to the thiophene π-spacer ligands in MH-12 and MH-13.

The emission spectra of all of the dyes display characteristic *λ*_emi_ in the region of 790–810 nm. It is seen that the *λ*_emi_ of IA-7 (806 nm) is red shifted when compared to that of MH-12 (796 nm) and MH-13 (803 nm). The perceived red shift is attributed to the presence of a strong electron donor in the IA-7 complex. The intersection of the absorption and fluorescence spectra generates the optical band-gap values. Their optical band gaps increase in the following order: MH-13 (1.82 eV) > MH-12 (1.85 eV) > IA-7 (1.88 eV) and the Stokes shifts are: MH-13 (249 nm), IA-7 (245 nm), and MH-12 (243 nm). Among the tested dyes, IA-7 exhibited the highest molar extinction coefficient, which translated into better population of the excitonic state and photocurrent density due to the existence of the strong and bulky electron donor ancillary ligand of TPA.

Cyclic voltammetry (CV) studies were performed for IA-7, MH-12 and MH-13 to investigate the thermodynamics of electron movement from the excited states of the dyes to the CB of TiO_2_ and the dye regeneration from the electrolyte redox couple to the oxidized dyes.^25^ The voltammograms are presented in the ESI (see ESI, Fig. S15†). The measurements were performed at 25 °C using a Vertex electrochemical workstation with a conventional three electrode configuration, comprising a platinum disc as a passive electrode, glassy carbon (GC) as the working electrode, and Ag/AgCl as the reference electrode. The potentials are reported *vs.* ferrocene as a standard reference at 100 mV s^−1^ scan rate using 0.1 M tetra-*n*-butyl ammonium hexafluorophosphate ((*n*-Bu)_4_N^+^(PF_6_)^−^) as a supporting electrolyte in acetonitrile under an argon atmosphere. From the voltammograms, it is observed that all of the dyes display two characteristic energy levels; the first is the ground state oxidation potential (GSOP) energy level in the range of −5.46 to −5.56 eV and the second is the excited state oxidation potential (ESOP) energy level at about −3.58 to −3.71 eV. The GSOP energy level was determined from the onset oxidation potential (*E*^Oxd^_Onset_) of the oxidation peak of the cyclic voltammogram, utilizing eqn (1).

1 GSOP = −[*E*^Oxd^_Onset_ + 4.7] eV

It is observed that IA-7 attained the lowest GSOP level value as a result of the existence of the bulky donor incorporating methoxy groups in the molecule, which lowered the GSOP level of the dye, taking it away from the CB of TiO_2_, and pushed the GSOP level far from the electrolyte potential. To study more precisely the probability of dye injection and regeneration processes, the excited state oxidation potentials (ESOPs) were computed from the optical bandgap (E_0–0_) and GSOP values by using eqn (2).

2 ESOP = [GSOP − *E*_0–0_] eV

**Fig. 3:**
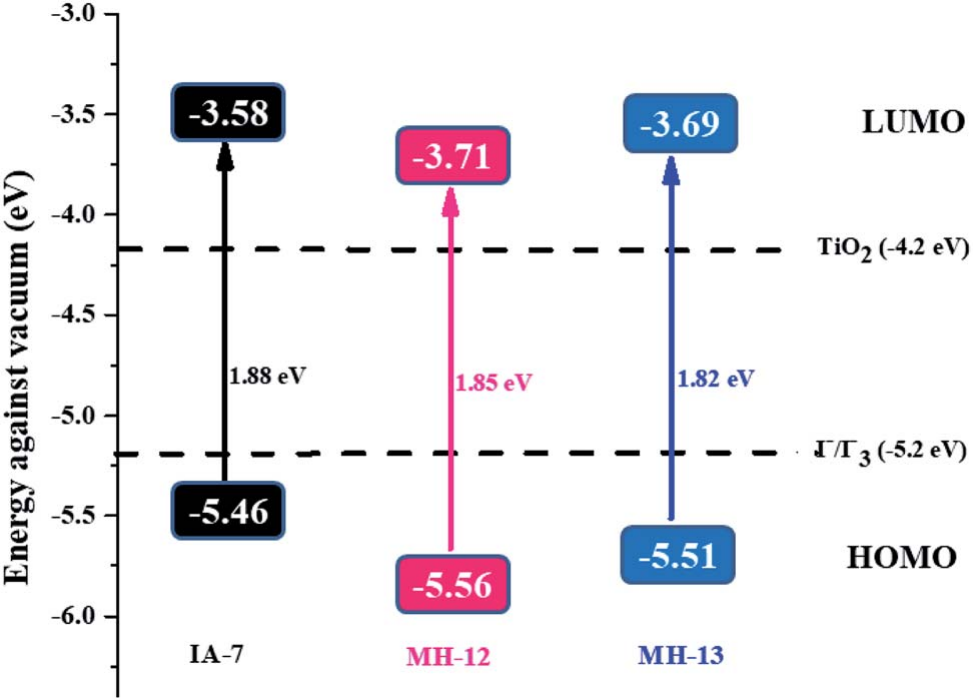
An energy diagram of the complexes IA-7, MH-12 and MH-13 compared to TiO_2_ and the *I*^−^/*I*_3_^−^ redox couple. GSOP and ESOP are reported together with the HOMO–LUMO energy gap (S0–S1).

It was noticeable that the data obtained for the GSOP and ESOP of the dyes (MH-12 and MH-13) were found to be the same as previously reported.^23^ The predicted GSOP levels of IA-7 (−5.46 eV), MH-12 (−5.56 eV), and MH-13 (−5.51 eV) were observed to be more negative than the *I*_3_^−^/*I*^−^electrolyte system potential.^26^ Meanwhile, the calculated ESOP levels were observed to be IA-7 (−3.58 eV), MH-12 (−3.71 eV) and MH-13 (−3.69 eV), which are energetically higher than the CB potential of TiO_2_. The photophysical and CV results are tabulated in Table 1. The energetic diagrams of complexes IA-7, MH-12 and MH-13 compared to TiO_2_ and the *I*^−^/*I*_3_^−^ redox couple are outlined in Fig. 3. From the results, the dyes IA-7, MH-12 and MH-13 are thermodynamically favorable for electron injection and dye regeneration in the fabricated devices. The electron injection free energy was observed to be in the order of IA-7 > MH-13 > MH-12 and this explains the superior thermodynamic stability for the IA-7 complex compared to the MH-12 and MH-13 complexes; this is due to the more thermodynamically favorable electron injection into the conduction band edge of TiO_2_, which decreases charge recombination in the fabricated devices.

### 3.3. Theoretical calculations

**Fig. 4:**
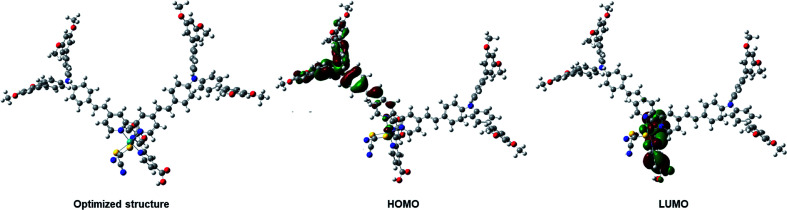
Optimized geometry and FMO energy levels for the IA-7 complex.

The FMO isosurface delocalization in the HOMO/LUMO of the IA-7 complex was evaluated *via* applying density functional theory (DFT) and time-dependent density functional theory (TD-DFT). All DFT and TD-DFT calculations were performed through North Carolina State University's High-Performance Computing utilizing GAUSSIAN 09 software to calculate the molecular geometry of the complex, electron delocalization over the HOMO/LUMO energy levels and vertical electronic excitation. DFT estimations were executed utilizing the hybrid functional B3LYP and LANL2DZ basis set. The resulting 3D optimized structures of the dyes with frontier molecular orbitals of the HOMO–LUMO isosurfaces are represented in Fig. 4. The optimized structure of the lowest energy conformer shows a carbon–carbon double bond with *E E*-conformation. As expected, from the HOMO energy levels, the electron density is predominantly delocalized on the bulky TPA units. However, the LUMO energy level is shifted from the electron donor to the electron acceptor units (the bipyridyl ligand incorporating the anchoring groups). The results from FMO revealed that dye IA-7 showed effective charge separation, which translated into greater ICT behavior in the IA-7 complex and high photovoltaic performance. On the other hand, TD-DFT studies were carried out for IA-7 in order to probe their electronic excitations. TD-DFT was performed on the optimized structure *via* the correlation functional B3LYP and the DGDZVP basis set. From TD-DFT calculations and GSOP and ESOP energy level calculations, clearly the theoretical results are in good agreement with the experimental data, and electron injection for the IA-7 complex is thermodynamically favorable as it′s ESOP, −3.46, is positively shifted compared to the calculated conduction band edge of TiO_2_ (−4.04 eV) as shown in Table 2.

**Table 2:** Calculated HOMO–LUMO energy levels and lowest TD-DFT excitation energies for IA-7 compared to TiO_2_ nanoparticles^a^

Calculated energy (eV), TD-DFT						Experimental (eV)
Dye	*E*_LUMO_	*E*_HOMO_	GSOP	ESOP	*E*_0–0_=(S_0_–S_1_)	*E*_0−0_^*b*^
IA-7	−3.087	−5.34	−5.34	−3.48	1.86	1.88
TiO_2_	−3.44	−7.16	−7.16	−4.04	3.12	3.20

a *E*_0–0_ = (S_0_–S_1_) = the lowest vertical excitation energy; GSOP = ground state oxidation potential = *E*_HOMO_; ESOP = excited state oxidation potential = *E*_LUMO_; *E*_0−0_^*b*^ = experimental energy gap calculated from the point of intersection for the absorption and emission spectra (CH_2_Cl_2_).

### 3.4. Photovoltaic performance of DSSCs

DSSCs were fabricated as reported^27^ and more specifics are given in the ESI.† Furthermore, photovoltaic and incident photon-to-current efficiency (IPCE) estimations were accomplished for solar cell devices, which were prepared using platinum-coated counter electrodes and TiO_2_-covered photoanodes, which were prepared by using 0.2 mM dye solutions in 1 : 1 : 1 acetonitrile, *tert*-butanol and DMSO solvents; the co-absorbent chenodeoxycholic acid (CDCA, 20 mM) was used in the solution, which acts as an anti-aggregation reagent.^28–31^ The dyes were absorbed on the mesoporous TiO_2_ electrode. The two electrodes were sealed together after adding redox electrolyte (Solaronix, Iodolyte HI-30) consisting of a solution of 0.6 M DMPII, 0.05 M I_2_, 0.1 M LiI and 0.5 M TBP in acetonitrile. The photovoltaic parameters of the fabricated DSSCs were specified under an Oriel solar simulator (AM 1.5 G, 100 mW cm^−2^) attached to a Keithley 2400 digital-source meter.^32^ The photocurrent efficiency (*η*) is given in eqn (3) as a function of different photovoltaic parameters and the incident light power (*P*_in_).^33^3
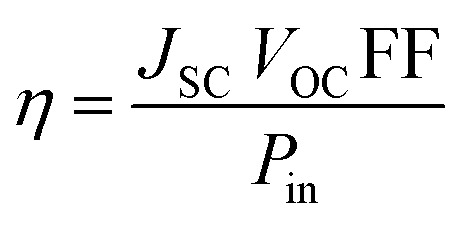


**Fig. 5:**
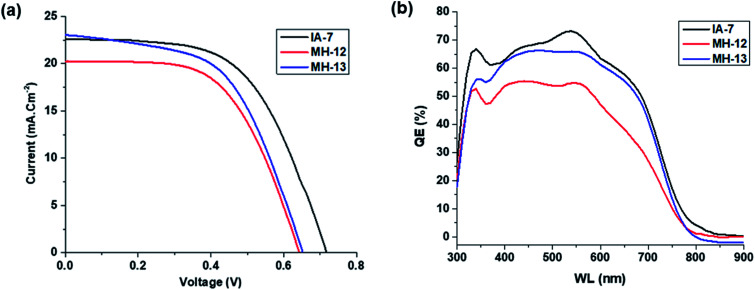
(a and b): Curves of solar cells sensitized with IA-7, MH-12, and MH-13 under 100 mW cm^−2^ light intensity: (a) *J*–*V* curves and (b) IPCE curves.

The photovoltaic performance of the fabricated DSSCs with IA-7, MH-12, and MH-13 is portrayed in Fig. 5a and the photovoltaic parameter information, such as *V*_OC_, *J*_SC_, FF and *η*, is outlined in Table 3. The results indicate that dye IA-7 achieved a maximum PCE of 8.86% (*J*_SC_ = 22.60 mA.cm^−2^, *V*_OC_ = 0.71 V, and FF = 56.19) compared to the PCE of 8.09% (*J*_SC_ = 20.23 mA.cm^−2^, *V*_OC_ = 0.65 V, and FF = 56.39) for MH-12 and PCE of 8.53% (*J*_SC_ = 22.97 mA.cm^−2^, *V*_OC_ = 0.66 V, and FF = 56.41) for MH-13. The *J*–*V* data indicate that the PCE of the IA-7 complex achieved a higher total conversion efficiency compared to MH-12 and MH-13; this is due to the open circuit voltage (*V*_OC_) of MH-13 being decreased by Δ*V*_OC_ = 0.06 V as compared with complex IA-7. The outstanding open circuit voltage (*V*_OC_) of the IA-7 complex is attributed to the presence of a strong electron rich donor (Bulky-TPA) motif as an ancillary ligand in the dye molecule, which substantially enhanced charge injection into the CB edge of TiO_2_, while the MH-12 and MH-13 ligands possess only π-spacers.

**Table 3:** Photovoltaic parameters of the fabricated DSSCs under the illumination of AM-1.5 solar light (100 mW cm^−2^)

Sensitizer (0.2 mM)	CDCA (mM)	*J*_SC_ (mA cm^−2^)	*V*_OC_ (V)	FF (%)	*η* (%)
IA-7	20	22.60	0.71	56.19	8.86
MH-12	20	20.23	0.65	56.39	8.09
MH-13	20	22.97	0.66	56.41	8.53

Furthermore, IPCE experiments were conducted using the same fabricated devices utilizing the QEX10 spectral response measurement system. IPCE quantitatively characterizes the amount of current that a cell will produce when irradiated with photons at a given wavelength in the solar spectrum and is determined by eqn (4), where *J*_SC_ is the photo-current density, *q* is the elementary charge, *λ* is the wavelength of incident irradiation, and *P*_0_ is the incident irradiation intensity.4
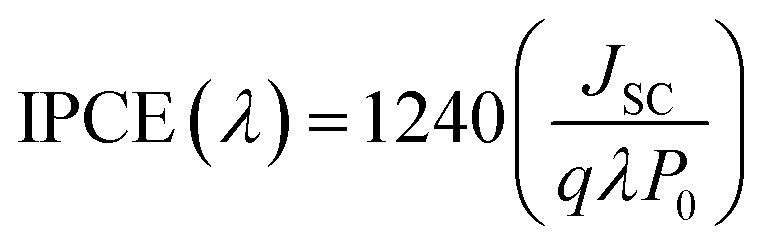


The IPCE curves of IA-7, MH-12 and MH-13 are depicted in Fig. 5b. It is obvious that the IPCE of IA-7 displays a broad band in the region of 350–700 nm with a maximum value of 73%, while MH-13 exhibits a broad band with a maximum value of 69% and MH-12 shows a wide band in the region of 375–600 nm with a maximum value of 55%. The superior IPCE of IA-7 translates into a higher PCE value of 8.86% compared to that of MH-13 (8.53%) and MH-12 (8.09%).

From the IPCE curves (Fig. 5b), it is evident that the response of complex IA-7 is superior and extends into the near infrared region more than that of MH-12 and MH-13. Moreover, the IPCE rises gradually until it reaches over 73% for complex IA-7 in the high energy region (570 nm), compared to MH-12 and MH-13, and these data are in good agreement with the UV-Vis spectrum data of these complexes.

### 3.5. Electrochemical impedance studies (EIS)

**Fig. 6:**
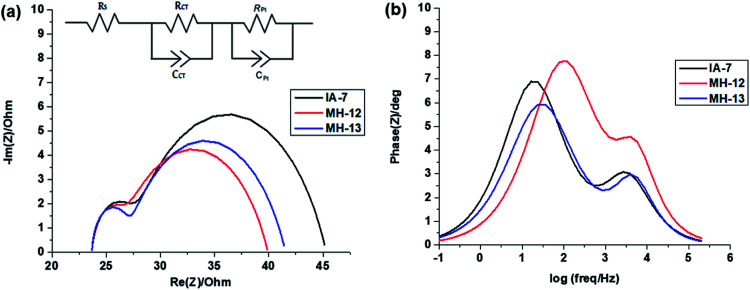
(a and b): Electrochemical impedance spectra: (a) Nyquist plots and (b) Bode plots for DSSCs sensitized with IA-7, MH-12, and MH-13.

The electrochemical impedance spectra were obtained from a Biologic SP-150 potentiated under illumination using a solar simulator. The EIS measurements were performed to understand the transportation of electrons and interfacial charge recombination behavior in the fabricated solar cells.^34–36^ The Nyquist and Bode plots of the fabricated DSSCs with IA-7, MH-12, and MH-13, respectively are provided in Fig. 6a and b. In the Nyquist plots shown in Fig. 6a, a higher frequency of the semicircle relates to the series ohmic resistance (*R*_S_), whereas the high-frequency region gives the interface capacitance (*C*_Pt_) and charge transport resistance (*R*_Pt_) at the counter electrode/electrolyte interface. Similarly, the low-frequency region is attributed to chemical capacitance (*C*_μ_) and the charge recombination (*R*_CT_) resistance at the TiO_2_/dye/electrolyte interface. The bigger the radius of the semicircle, the higher the charge recombination resistance from TiO_2_ to the *I*_3_^−^/*I*^−^ electrolyte will be.^37,38^ However, a decrease in the radius of the semicircle was observed in the order of MH-12 > MH-13 > IA-7. This trend demonstrates that the recombination at the TiO_2_/dye/electrolyte interface is IA-7 > MH-13 > MH-12, and the high recombination resistance of IA-7 compared to the other reported dyes enhances electron injection into the mesoporous TiO_2_ nanoparticles and so increases the overall efficiency of DSSCs. The observed results are consistent with the achieved PCE values of IA-7, MH-12 and MH-13.

The Bode frequency plots arise from electron transport at the TiO_2_/electrolyte interface. The peaks from the MH-12 and MH-13 complexes in the Bode phase plots in Fig. 6b are at higher frequencies than that from the IA-7 complex, indicating that the electron lifetime of IA-7 is higher than that of the MH-12 and MH-13 complexes. The effective lifetime (*τ*_eff_) of electrons injected into the CB of TiO_2_ was estimated as a function of the corresponding frequency peak (*f*) in the Bode phase plot, as given in eqn (5)_._5
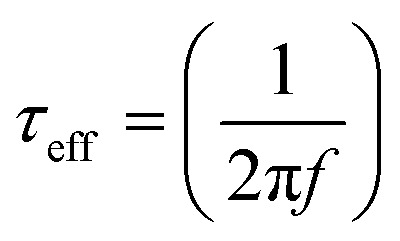


The effective lifetime *τ*_eff_ is calculated for the IA-7, MH-12, and MH-13 sensitized devices to be IA-7 (8.93 ms) > MH-13 (5.87 ms) > MH-12 (2.0 1 ms), respectively. On a fundamental level, a longer electron lifetime indicates better suppression of back reactions between the injected electrons and the electrolyte, which ordinarily leads to improvement of the *V*_OC_^39,40^ as shown in Table 1. The increase in *V*_OC_ value for the IA-7 complex could be caused by the retardation between injected electrons and oxidized species in the electrolyte. Besides, these results clearly show the great effect of donor and π-spacer antenna ligands on the electron recombination processes between the electrolyte species and electron transfer into the TiO_2_ semiconductor film. Hence, the decrease in *V*_OC_ for the MH-12 and MH-13 cells can be explained by their faster recombination relative to that of complex IA-7.

## 4. Conclusions

In summary, we have designed and developed a novel Ru(ii) photosensitizer, denoted as IA-7, based on a bulky TPA antenna ligand for DSSC applications. The spectrophotometric, electrochemical, and device performance response studies of IA-7 were compared to our previously reported MH-12 & MH-13 complexes. Interestingly, the complex IA-7 achieved a maximum power conversion efficiency (PCE) of 8.86% (*J*_SC_ = 22.60 mA cm^−2^) compared to 8.09% for MH-12 and 8.53% for MH-13. The device performances of all dyes displayed an enhanced *V*_OC_ value for the complex IA-7, which can be clarified based on its capacity to suppress charge recombination processes. The superior PCE of IA-7 can be assigned to the high molar absorptivity of IA-7 for the MLCT peak and the superiority in light harvesting through the bulky electron donor antenna ligand compared to the MH-12 & MH-13 dyes, which incorporate π-spacer antenna ligands. Conclusively, the donor strength in the structural motif of the dye is a key factor in enhancing the total conversion efficiency in DSSCs.

## Conflicts of interest

There are no conflicts to declare.

## Supplementary Material

RA-010-C9RA06150A-s001
