# Supplementary material for: The molecular engineering, synthesis and photovoltaic studies of a novel highly efficient Ru(ii) complex incorporating a bulky TPA ancillary ligand for DSSCs: donor versus π-spacer effects
Source: RSC Adv. 2020 Jan 2;10(1):610–9. doi: 10.1039/c9ra06150a (PMC9047394; doi:10.1039/c9ra06150a)
Supplement: RA-010-C9RA06150A-s001 [file RA-010-C9RA06150A-s001.pdf]

Electronic Supplementary Material (ESI) for RSC Advances.
This journal is © The Royal Society of Chemistry 2019

Electronic Supplementary Information

Molecular Engineering, Synthesis and Photovoltaic Studies of Novel Highly Efficient Ru (II) Complex Incorporating Bulky TPA Ancillary Ligand for DSSCs: Donor Versus π-Spacers Effect

*Islam M. Abdellah[1] and Ahmed El-Shafei[2, *]*

[1]*Department of Chemistry, Faculty of Science, Aswan University, Aswan, 81528, Egypt*
[2] *Polymer and Color Chemistry Program, North Carolina State University, Raleigh, 27606, USA*

***Corresponding Author**: Ahmed El-Shafei, NC State University, Department of TECS, Raleigh, NC, USA, E-Mail: Ahmed_El-Shafei@ncsu.edu.

1. Device fabrication

1.1. Electrode Preparation and Device Fabrication

Fluorine-doped tin oxide (FTO) coated glasses (2.2 mm thickness, sheet resistance of 8 Ω/cm^2, TEC, Pilkington) were washed with detergent, water, acetone and ethanol, sequentially. After this FTO glass plates were immersed into a 40 mM aqueous $TiCl_4$ solution at 70 °C for 30 min and washed with water and ethanol. Thin layer (8-12 µm thick) of TiO_2 (Solaronix, Ti-Nanoxide T/SP) was deposited (active area, 0.18 cm^2) on transparent conducting glass by squeegee printing followed by drying at 350 °C for 10 min and curing at 500 °C for 30 min. Next, after drying the electrodes, scattering layer (5 µm thick) TiO_2 particles (Solaronix, Ti-Nanoxide R/SP) were printed onto the already deposited TiO_2 layer. The TiO_2 electrodes were heated under an air flow at 350 °C for 10 min, followed by heating at 500 °C for 30 min. After cooling to room temperature, the TiO_2 electrodes were treated with 40 mM aqueous solution of $TiCl_4$ at 70 °C for 30 min and then washed with water and ethanol. The electrodes were heated again at 500 °C for 30 min and left to cool to 80 °C before dipping them into the dye solution. The dye solutions (0.2 and 0.3 mM) were prepared in 10 mL 1:1:1 acetonitrile, DMSO and *tert*-butanol. Chenodeoxycholic acid (CDCA) was added at a concentration of 20 mM. The electrodes were

immersed in the dye solutions and then kept at 25 °C for 20 hours to adsorb the dye onto the TiO_2 surface.

For preparing the counter electrode, pre-cut TCO glasses were washed with water followed by 0.1M HCl in EtOH, and sonication in acetone bath for 10 min. These washed TCO were then dried at 400 °C for 15 min. Thin layer of Pt-paste (Solaronix, Platisol T/SP) on TCO was printed and the printed electrodes were then cured at 450 °C for 10 min. The dye sensitized TiO_2 electrodes were sandwiched with Pt counter electrodes and the electrolyte (Solaronix, Iodolyte HI-30) was then injected into the cell, while the two electrodes were held together with the clips.

2. Synthesis

2.1. Synthesis of 2, 2'-Bipyridinyl-4, 4'-dicarboxylic acid

The 2, 2'-Bipyridinyl-4, 4'-dicarboxylic acid was synthesized according to published procedure [3]. In a three neck round bottom flask 4,4'-Dimethyl-2,2'-bipyridine (3 g, 16.3 mmol) was gradually added to a stirred solution of sodium dichromate ($Na_2Cr_2O_7$) (10.9g, 36.5 mmol) in concentrated sulfuric acid (H_2SO_4) (93%, 45 ml) and stirred for 30 min at 75°C. After 30 min, the dark green reaction mixture was poured into cold water forming a light-yellow precipitate. The precipitate was then filtered under vacuum and dissolved in 10 % aq. Sodium hydroxide. The product was crystallized by slowly acidifying the solution with 10 % aq. hydrochloric acid at a pH of 2. The precipitation and acidification process were repeated three times to obtain the white chromium free precipitates of 2, 2'-Bipyridinyl-4, 4'-dicarboxylic acid (2.56g, 90%).

3. Spectral Data

3.1. High Resolution Mass Spectral data

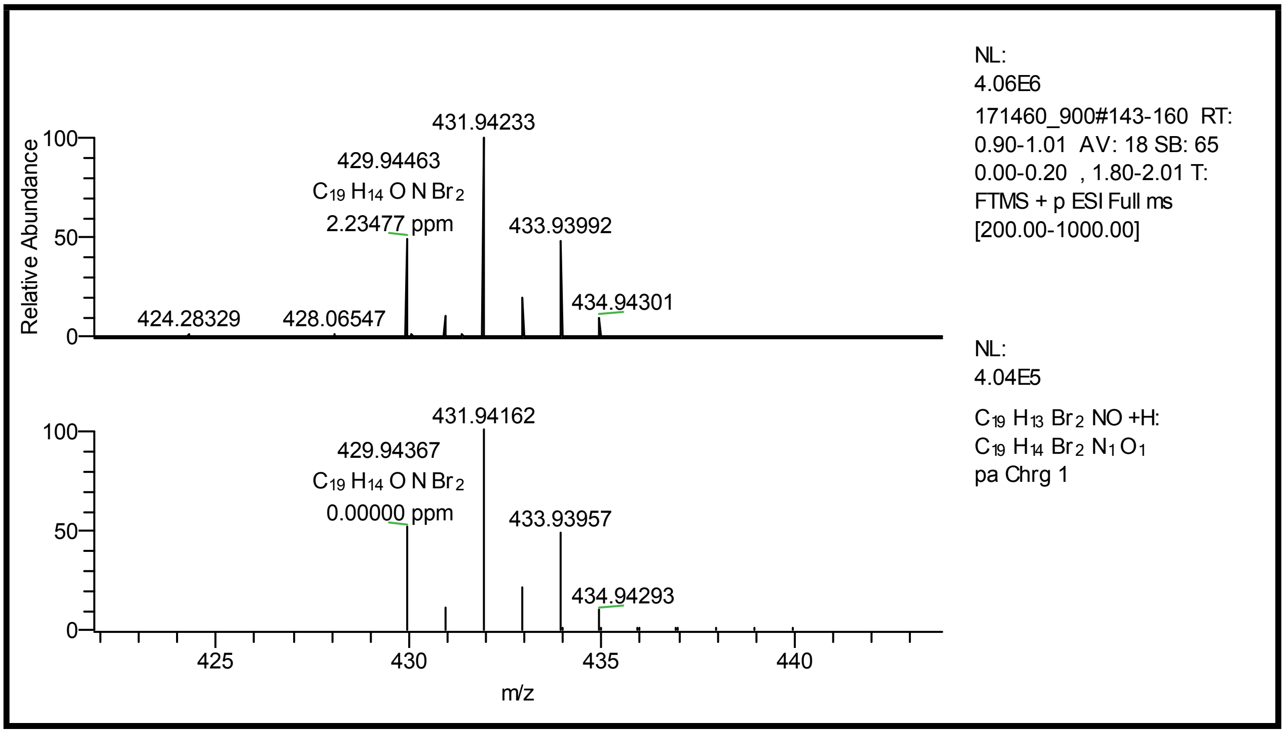

Fig. **S1.** Experimental and Theoretical Isotopic Distribution for **1**; **[M+H]**$^+$

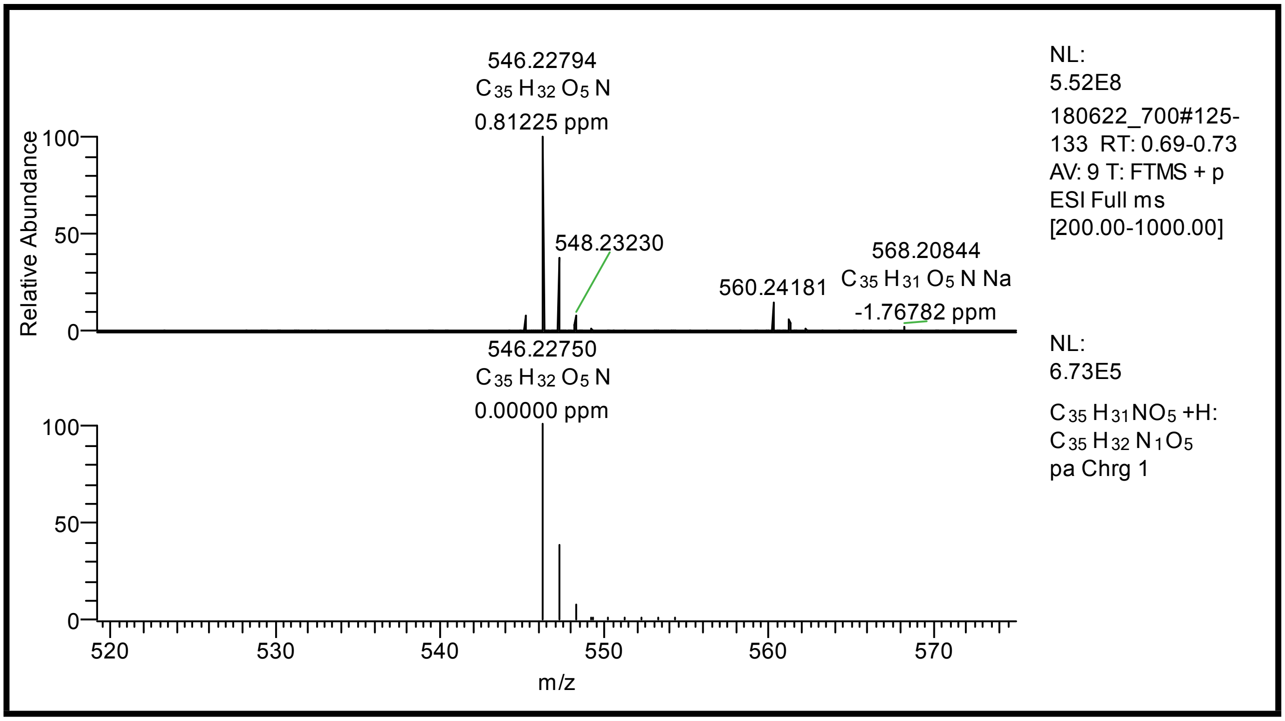

Fig. **S2.** Experimental and Theoretical Isotopic Distribution for **2**; **[M+H]**$^+$

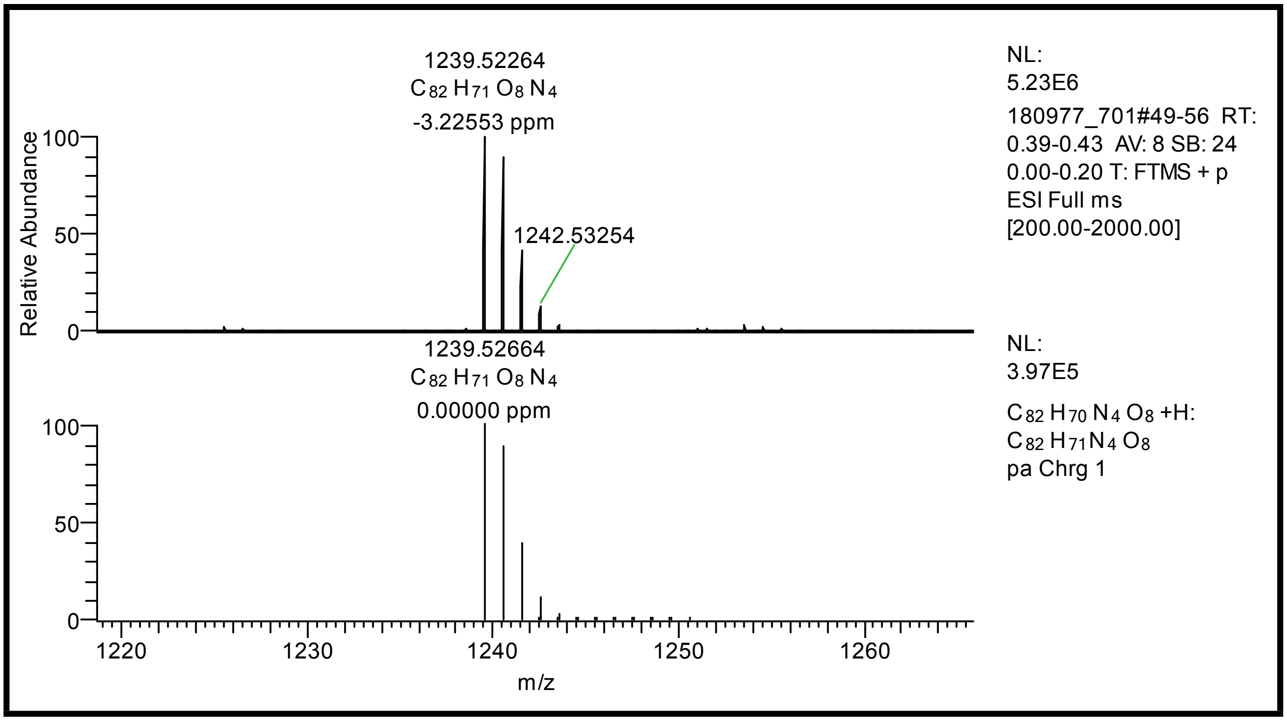

Fig. **S3.** Experimental and Theoretical Isotopic Distribution for **3**; **[M+H]**$^+$

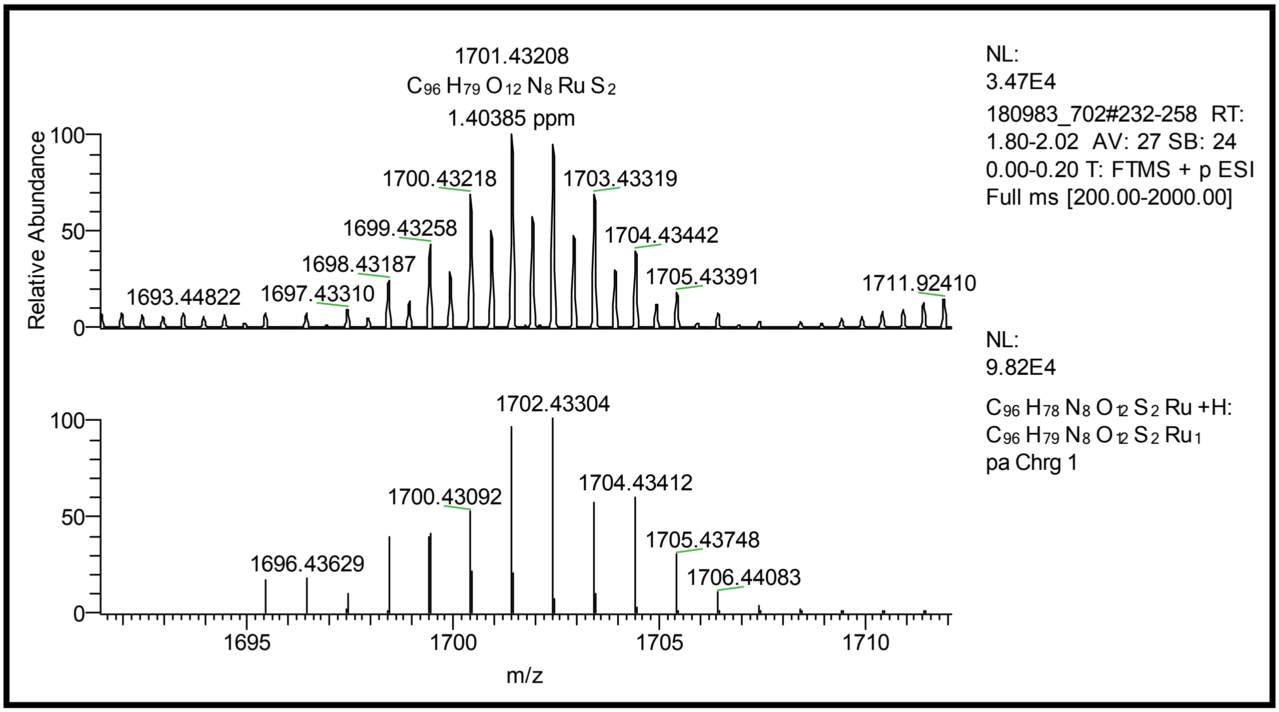

Fig. **S4.** Experimental and Theoretical Isotopic Distribution for **IA-7**; **[M+H]**$^+$

3.2. FT-IR Spectral Data

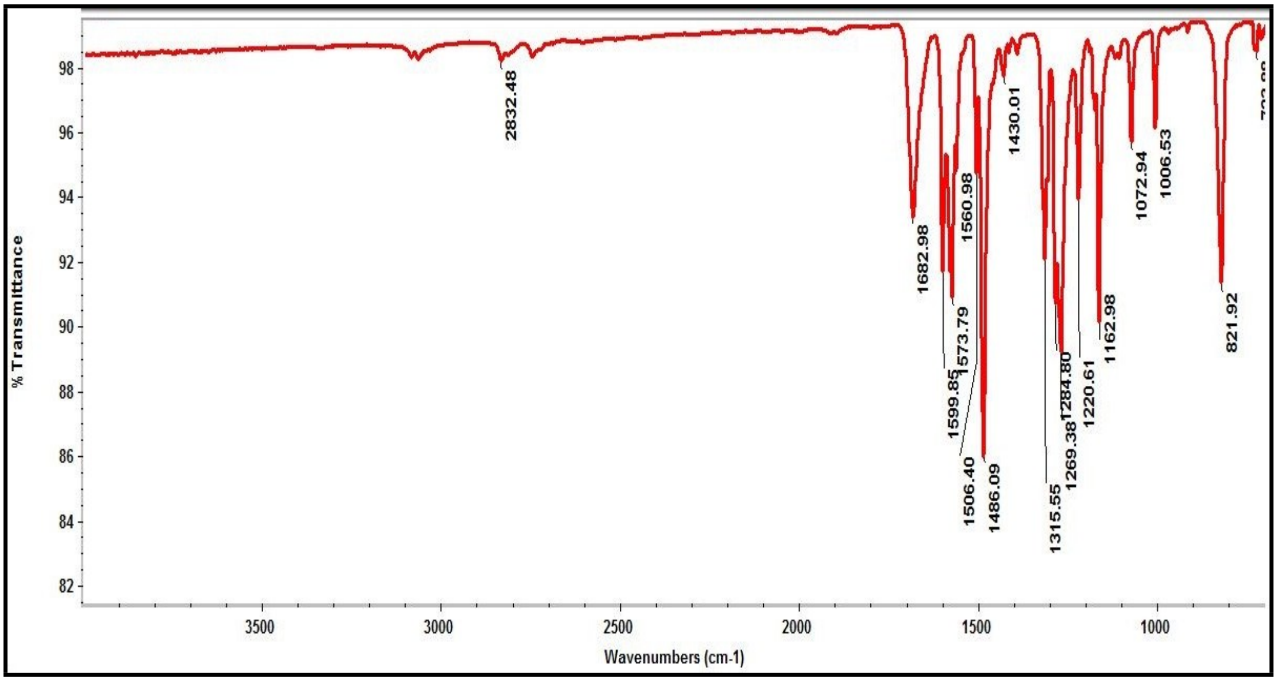

Fig. **S5.** FTIR spectra for compound **1**

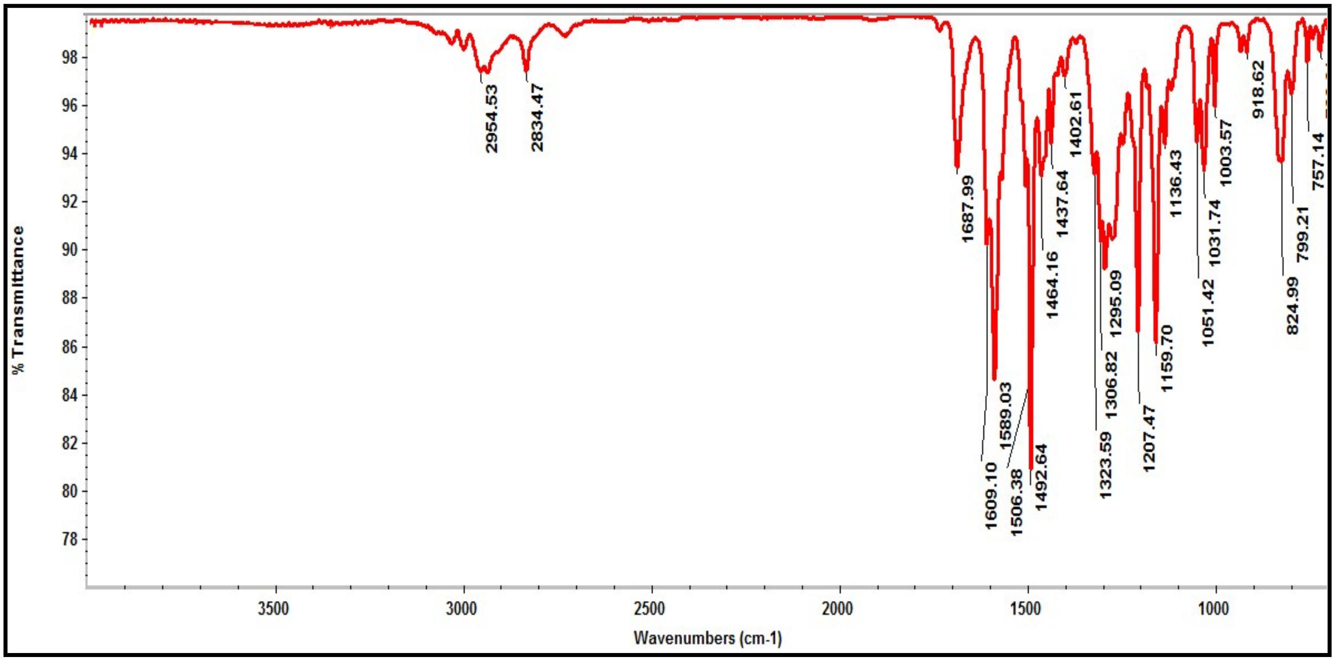

Fig. **S6.** FTIR spectra for compound **(2)**

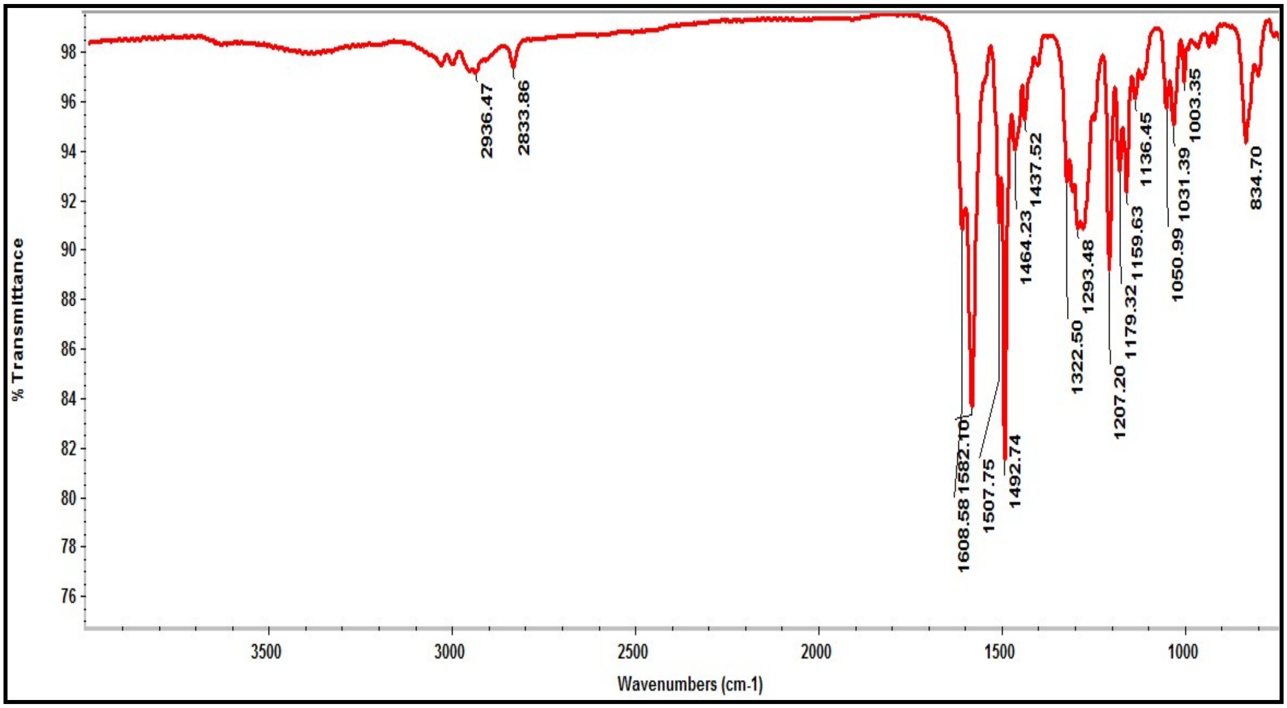

Fig. **S7.** FTIR spectra for compound **(3)**

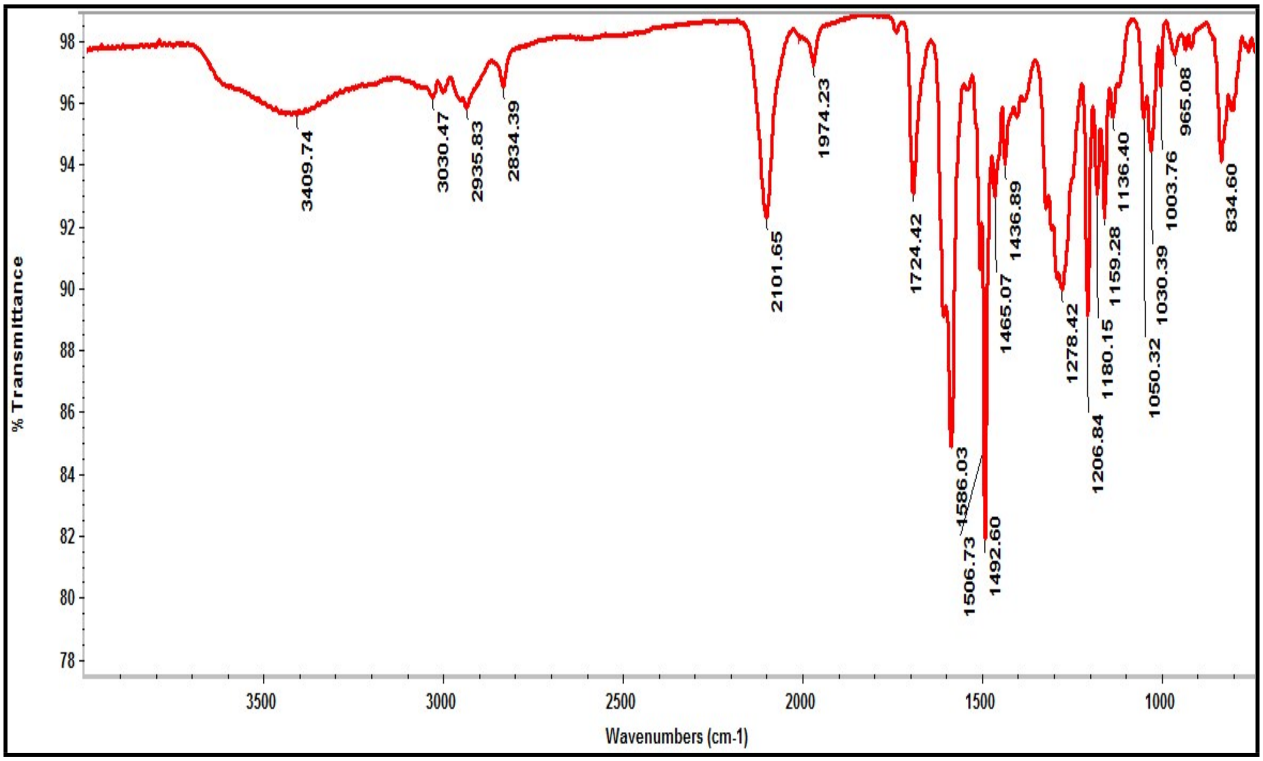

Fig. **S8.** FTIR spectra for dye **(IA-7)**

3.3. ^{1}H-NMR Spectral Data

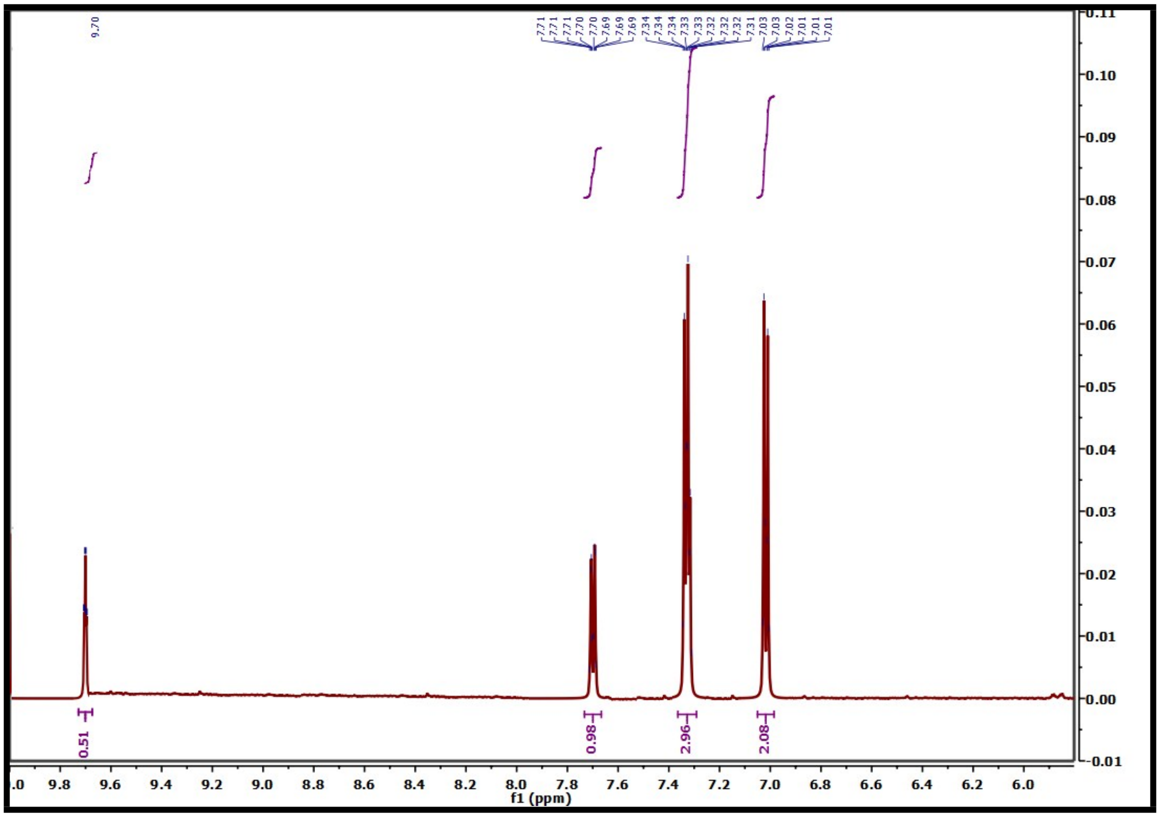

Fig. **S9.** ^{1}H-NMR spectra of compound **1**

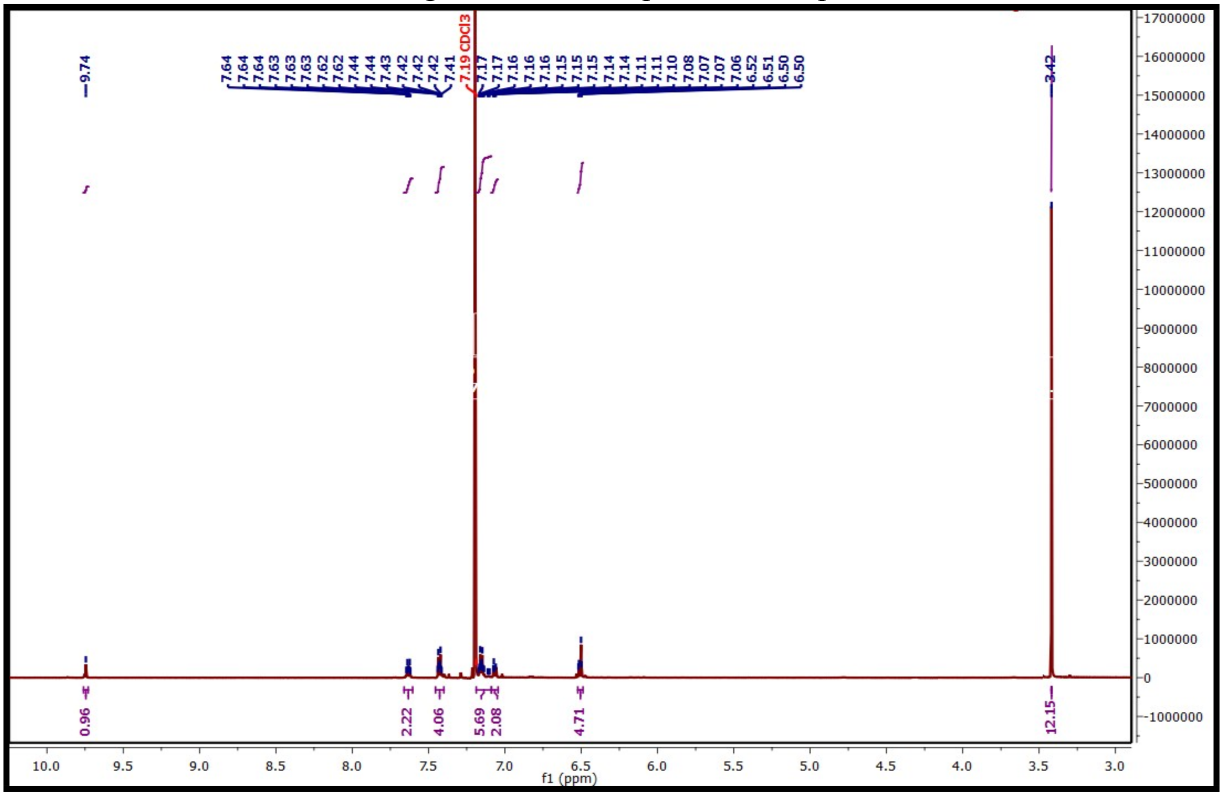

Fig. **S10.** ^{1}H-NMR spectra of compound **2**

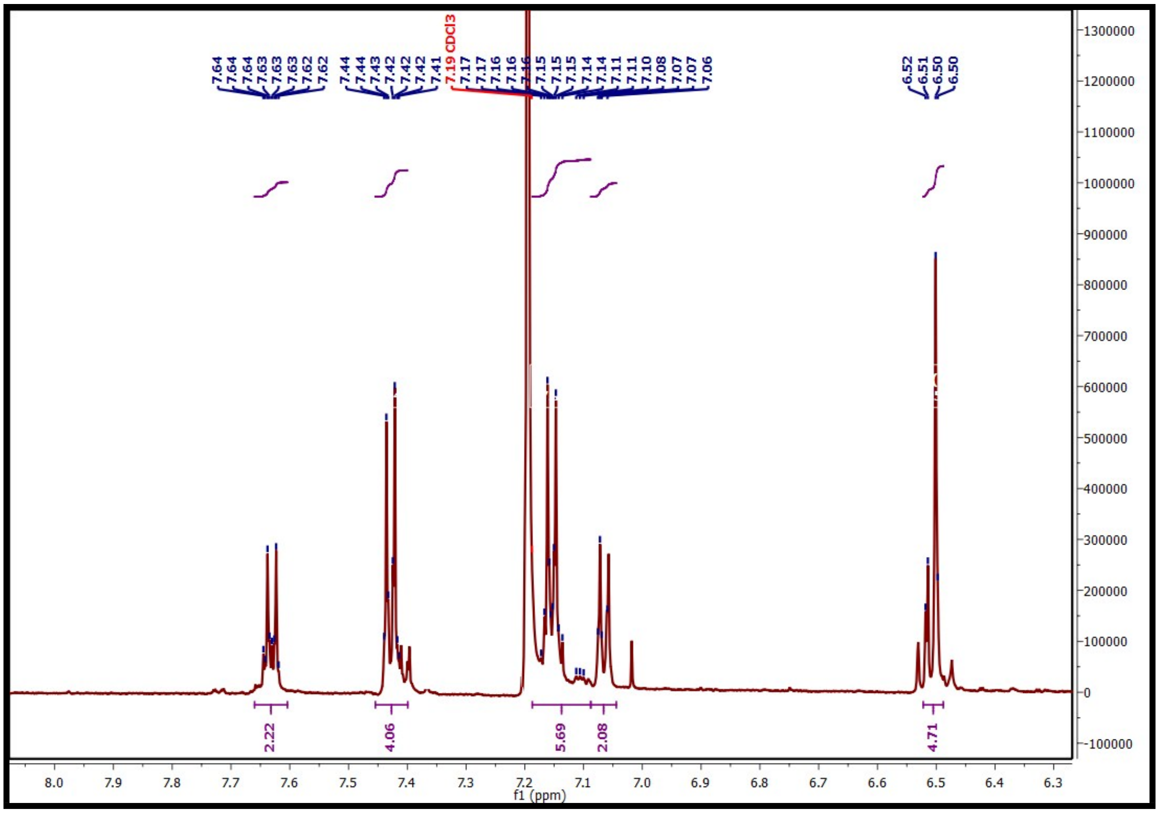

Fig. **S11**. ^{1}H-NMR spectra of compound **2 (Aromatic Zoom)**

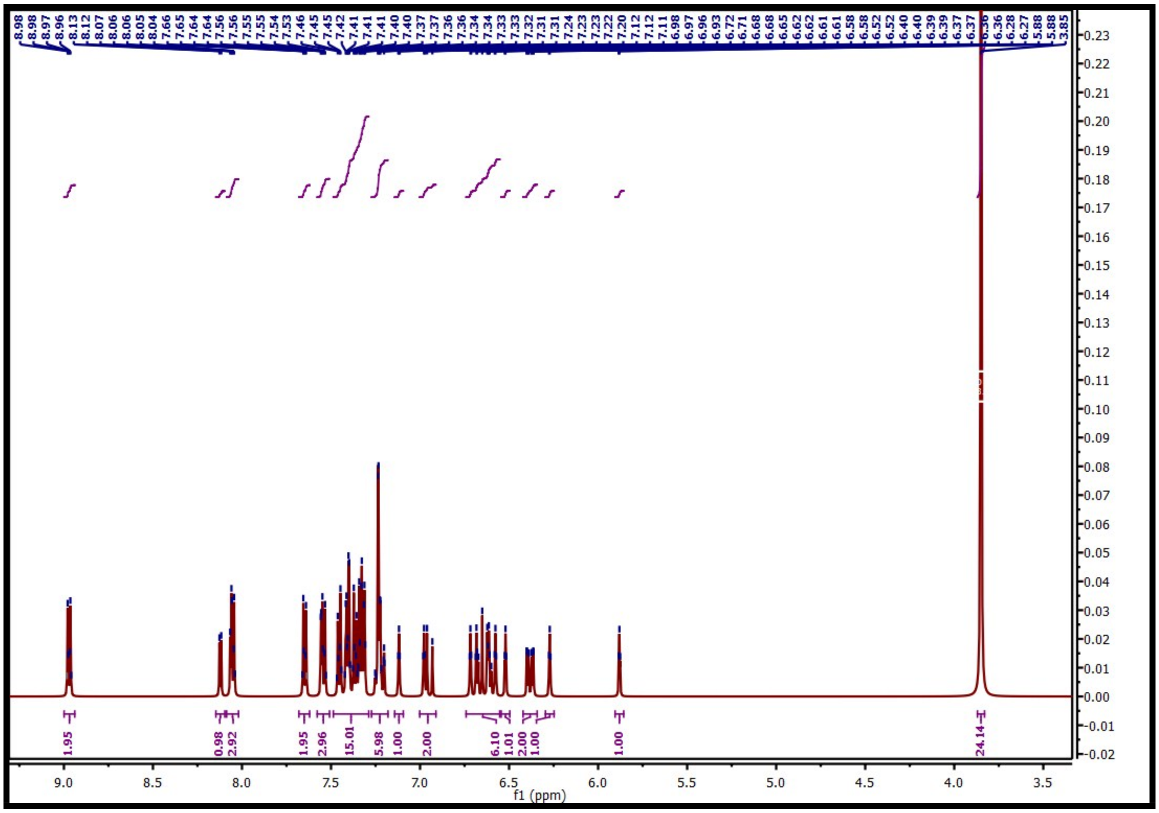

Fig. **S12**. ^{1}H-NMR spectra of compound **3**

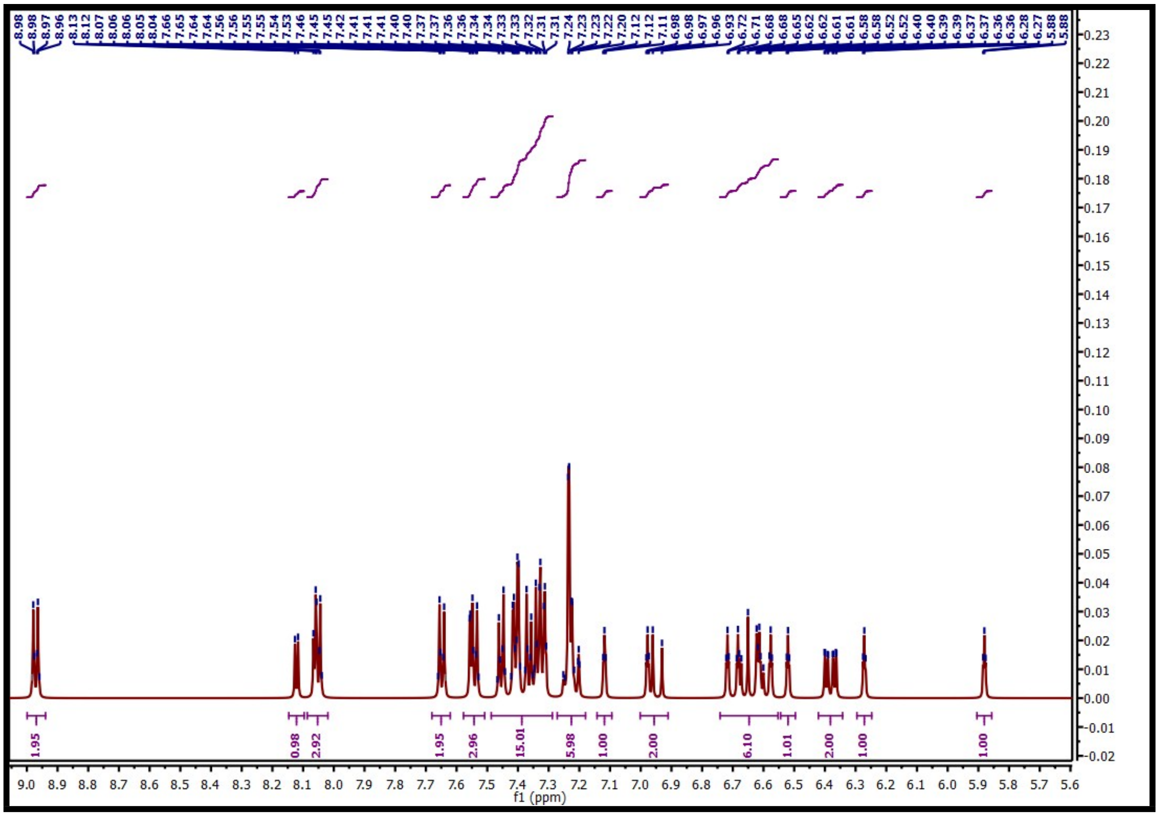

Fig. **S13.** ^{1}H-NMR spectra of compound **3 (Aromatic Zoom)**

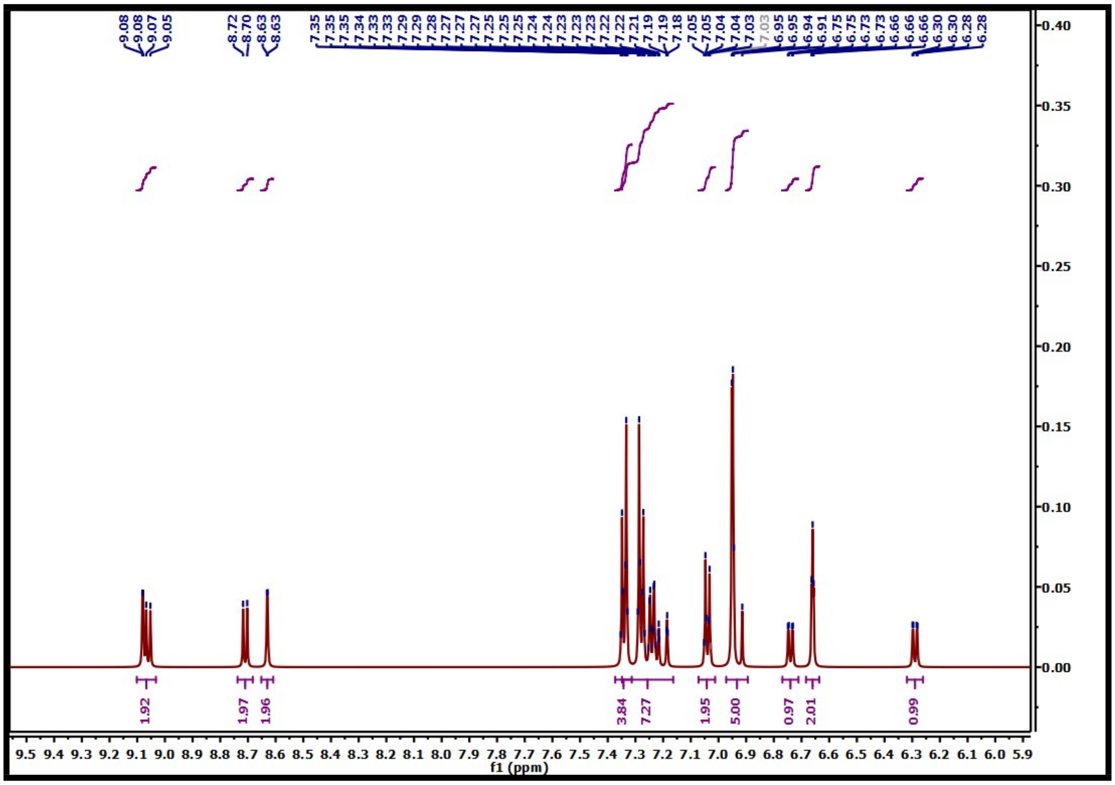

Fig. **S14.** ^{1}H-NMR spectra of dye **IA-7**

4. Cyclic Voltammograms for IA-7 complex

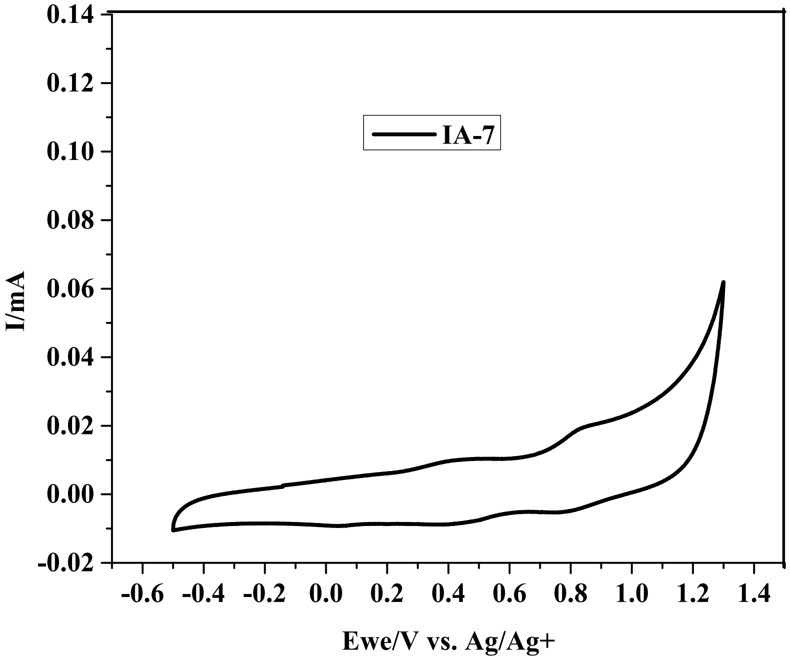

Fig. **S15.** Cyclic Voltammograms of **IA-7** complex
